# Non-Albumin Proteinuria (NAP) as a Complementary Marker for Diabetic Kidney Disease (DKD)

**DOI:** 10.3390/life11030224

**Published:** 2021-03-10

**Authors:** Jaehyun Bae, Young Jun Won, Byung-Wan Lee

**Affiliations:** 1 Division of Endocrinology and Metabolism, Department of Internal Medicine, Catholic Kwandong University College of Medicine, International St. Mary’s Hospital, Incheon KS006, Korea; baejh0419@ish.ac.kr (J.B.); yjwon@ish.ac.kr (Y.J.W.); 2 Division of Endocrinology and Metabolism, Department of Internal Medicine, Yonsei University College of Medicine, Seoul KS013, Korea

**Keywords:** diabetic kidney disease, tubular injury, non-albumin proteinuria

## Abstract

Diabetic kidney disease (DKD) is one of the most common forms of chronic kidney disease. Its pathogenic mechanism is complex, and it can affect entire structures of the kidney. However, conventional approaches to early stage DKD have focused on changes to the glomerulus. Current standard screening tools for DKD, albuminuria, and estimated glomerular filtration rate are insufficient to reflect early tubular injury. Therefore, many tubular biomarkers have been suggested. Non-albumin proteinuria (NAP) contains a wide range of tubular biomarkers and is convenient to measure. We reviewed the clinical meanings of NAP and its significance as a marker for early stage DKD.

## 1. Introduction

Diabetic kidney disease (DKD) is one of the most frequent complications of diabetes mellitus (DM), and the leading cause of end-stage renal disease (ESRD) [1]. It occurs in 20%~40% of patients with DM, and is diagnosed by the presence of persistently increased albuminuria and/or decreased estimated glomerular filtration rate (eGFR) [2]. DKD is also known to be associated with the increased risk of cardiovascular disease and mortality [3].

Screening for DKD is recommended for all type 2 DM patients and type 1 DM patients with a duration of ≥5 years on the basis of annual measurements of albuminuria and eGFR [2]. In general, albuminuria is known to reflect glomerular damage [4], and is considered to be a marker for early stage DKD, whereas a decrease in eGFR is usually considered to reflect advanced stages of DKD [5]. However, the pathophysiology of DKD encompasses a wide range of mechanisms including tubular change, as well as glomerular damage [6,7]. Therefore, albuminuria, a marker of glomerular damage, may be insufficient in detecting early stages of DKD. In fact, previous studies have reported that DKD may progress without significant albuminuria [8,9].

In this context, studies on alternative DKD markers other than albuminuria and eGFR have been conducted. In particular, there is accumulating evidence on the importance of urinary proteins other than albumin as markers for early stage DKD [10,11,12]. Therefore, non-albumin proteinuria (NAP), which is composed of these urinary proteins, is an attractive candidate for early-DKD markers in terms of its clinical significance and convenience [13,14]. In this article, we will review what status NAP reflects in the course of DKD and its clinical significance.

## 2. What NAP Reflects in DKD

### 2.1. Pathogenesis and Structural Changes of DKD

It is well-known that hyperglycemia is a central factor in the development and progression of DKD. This is supported by various studies which show that intensive glycemic control significantly lowers the prevalence of DKD and delays its progression [15,16,17,18]. However, factors other than hyperglycemia are also believed to play an important role in the pathogenesis of DKD. The activation of the renin–angiotensin–aldosterone system (RAAS), accumulation of advanced glycation end products (AGEs), generation of mitochondrial reactive oxygen species (ROS), oxidative stress, and presence of low-grade inflammation are representative of those factors [19,20,21,22,23,24].

As can be inferred from the existence of various pathogenic factors, structural changes in DKD are presented as various alterations in kidneys [7]. The classification developed by the Renal Pathology Society showed these in an organized form, as presented in Table 1 and Table 2 [25].

Conventionally, investigations into DKD have focused on changes in glomerular structure. Thickening of the glomerular basement membrane is considered as the earliest structural change in DKD [26], subsequently loss of endothelial fenestration, effacement of the podocyte foot process, and later mesangial expansion develop. In the later stages of DKD, mesangial expansion progresses, subendothelial accumulation of proteins and the formation of glomerular microaneurysms result, and the subsequent coalescence into glomerulosclerosis occurs [7,26]. This traditional glomerular model is undoubtedly a well-investigated model that reflects the major characteristics of DKD. However, cases of renal insufficiency without such glomerular changes have been reported in a considerable portion of patients with DM [27,28].

Recently, changes in the renal tubule, especially in the proximal tubule, have gained attention as another major feature of DKD, and are expected to provide a new perspective in understanding early stage DKD. In DKD, structural changes in the renal tubule appear mainly in the form of tubular atrophy, peritubular capillary rarefaction, and interstitial fibrosis [29]. These changes may appear as subsequent changes after glomerulopathy, but can also be triggered by their own pathological mechanisms.

One of the most representative mechanisms is hypoxia. The proximal tubule is vulnerable to hypoxia because it requires significant energy and oxygen to maintain its reabsorptive functions. Notably, in diabetic conditions, the proximal tubule is at a high risk of exposure to hypoxia because of the following factors: increased oxygen consumption, impaired oxygen utilization, and decreased oxygen supply [30]. In DM, sodium-glucose cotransporter-2 (SGLT-2), the major contributor to renal glucose reabsorption, is usually overexpressed [31]. To maintain its increased activity of glucose reabsorption, the maintenance of the electrochemical gradient for sodium is also required, which demands the increased consumption of oxygen in Na^+^/K^+^ ATPase [32]. Furthermore, renal gluconeogenesis is increased in patients with DM [33], which also demands oxygen usage. In addition to the increase in oxygen consumption due to its metabolic alterations of the proximal tubule, DM also damages the structure and function of mitochondria [34], causing the inhibition of aerobic energy production. Finally, similarly to the glomerular changes, endothelial injury and peritubular capillary rarefaction caused by DM pathology reduces the oxygen supply to the proximal tubule [35].

Factors other than hypoxia can also cause tubular changes in DM. The activation of RAAS damages renal tubules [36] along with the glomerulus. Increased delivery of growth factors such as transforming growth factor-β, epidermal growth factor, and insulin-like growth factor-1 to renal tubules under diabetic conditions induces tubulointerstitial fibrosis in diabetic kidneys [30,37,38]. These pathologic factors of tubular injury are also associated with other chronic diseases such as non-alcoholic fatty liver (NAFLD) and hypertension. Therefore, type 2 DM patients who are likely to have comorbidities are at a relatively high risk of developing tubular injury. In order to understand DKD more accurately, it is necessary to pay more attention to both tubulopathy and glomerulopathy.

### 2.2. Albuminuria and NAP

#### 2.2.1. Albuminuria

Albuminuria is assessed by the urinary albumin-to-creatinine ratio (UACR) in a spot urine sample and abnormally elevated albuminuria is defined as UACR ≥ 30 mg/g [2]. Since there is generally biological variability in measurements of urinary albumin excretion, DM patients with UACR ≥ 30 mg/g in two of three samples collected within six months are considered to be albuminuric or to have DKD. In DM patients with albuminuria, the majority of albumin in their urine is excreted through the trans-glomerular passage [39]. The major mechanism of diabetic albuminuria is the impairment of the selective permeability of the glomerulus, caused by glomerular endothelial dysfunction [40]. Damage to the glycocalyx on the surface of the endothelium seems to be a significant pathologic change for albuminuria. Therefore, albuminuria is considered to be a marker of glomerular damage. Undoubtedly, it is a well-established marker of DKD, with the presence of albuminuria being proven to be associated with all-cause mortality and cardiovascular mortality as well as kidney outcomes [3,41]. Based on the classical view of the natural history of DKD (Table 3) [5,42], it is also considered to be an early marker of DKD, appearing prior to eGFR reduction.

However, in recent years, questions have been raised as to whether albuminuria is an indicator that can sufficiently reflect the early stage of DKD. There have been considerable cases which were reported to be inconsistent with the classical view of the natural history of DKD. Among DM patients with reduced eGFR, defined as eGFR < 60 mL/min/1.73 m^2^, patients without albuminuria were reported to present in a proportion of approximately 23–55% [43,44,45,46,47]. The portion of DKD without albuminuria, i.e., normoalbuminuric DKD, tended to be higher in patients with type 2 DM compared to those with type 1 DM.

Therefore, albuminuria, a marker of glomerular damage, may be insufficient to reflect early stage DKD. It has limitations with respect to reflecting complex mechanisms of DKD, particularly the tubular injury described above. The need for complementary markers to albuminuria for early stage DKD has been raised, and researchers have focused on urinary proteins other than albumin.

#### 2.2.2. NAP and Tubular Injury

To complement the limitation of albuminuria in reflecting the early stage of DKD, various urinary biomarkers have been suggested. Since albuminuria is a marker for glomerular damage, biomarkers which reflect other pathologic mechanisms of DKD would be acceptable as the complementary DKD marker to albuminuria. As a result, biomarkers of tubular injury have gained attention for decades. Tubular injury markers such as alpha-1 micro-globulin (A1M), liver-type fatty acid–binding protein (L-FABP), N-acetyl-beta-D-glucosaminidase (NAG), and kidney injury molecule-1 (KIM-1) have been reported to be associated with DKD.

A1M is a small molecular weight protein which usually passes through the glomerulus and is reabsorbed by proximal tubular cells [48]. The destruction of tubular cells by any stimulus can result in an increase of urinary A1M that has been positively correlated with UACR and negatively correlated with eGFR in patients with DM [49,50]. In addition, it has also been correlated with the duration and severity of DM, which demonstrates its significance as an indicator of DKD [50]. In a cross-sectional study published in 2010, 27.9% of normoalbuminuric type 2 DM patients exhibited increased urinary A1M levels [51]. This report demonstrated the usefulness of A1M as an early biomarker for DKD.

L-FABP is a low molecular weight protein expressed in the proximal tubule and liver [10]. An experimental model which was reported in 2004 suggested that urinary L-FABP reflected tubular stress, such as protein overload. Later, it was also interpreted as being associated with tubular hypoxia [11]. Urinary L-FABP reflected the severity of DKD accurately, and was also associated with the progression of DKD [10,52,53]. Elevated urinary L-FABP could occur at an early stage of DKD, even before albuminuria [52,53,54,55].

NAG, a lysosomal enzyme in proximal tubule epithelial cells [56], is also a well-studied biomarker of tubular injury. Urinary NAG is very sensitive to tubular damage [12,57], and has been reported to exhibit higher sensitivity and specificity than albuminuria and creatinine clearance in patients with DM [58]. Interestingly, NAG is significantly associated with various glycemic parameters such as hyperglycemia and glycemic variability [57,59,60], and is a significant predictor of carotid artery atherosclerosis in DM [61,62]. These data findings suggest that urinary NAG reflects the severity of DM and its vascular complications.

KIM-1 is a transmembrane glycoprotein that is mainly expressed when the proximal tubule is damaged or under dedifferentiation after injury [63]. Urinary KIM-1 is known to be specific for kidney injury. It has been found to be correlated with UACR [64,65] and can be detected in DM patients with normo-albuminuria [66], like the above-mentioned biomarkers. A previous large-population prospective study showed a correlation between urinary KIM-1 and UACR, duration of DM, glycemic control, and long-term mortality, but the authors concluded that KIM-1 did not confer additional prognostic information regarding albuminuria [67].

In addition to the above-mentioned biomarkers, other tubular markers including retinol binding protein, beta-2 micro-globulin (B2M), and neutrophil gelatinase associated lipocalin (NGAL) have also been studied, and their association with DKD has been reported [10]. As well as the urinary biomarkers, serum or plasma markers such as uric acid and indoxyl sulfate, a uremic toxin, have been introduced as indicators of tubular injury [68,69]. However, so far, a single biomarker or a set of several tubular injury markers has not been accepted as a screening tool for DKD, compared to albuminuria. The most important reason was that these alternative markers did not have as much data as albuminuria. Furthermore, these biomarkers are inconvenient and expensive to measure in routine clinical settings.

In that context, NAP can be considered as a useful tubular marker because it contains a wide range of urinary proteins that reflect tubular injury and is easy to measure. In a cross-sectional analysis of type 2 DM patients, NAP is well-correlated with urinary tubular markers including L-FABP KIM-1 and NGAL [13]. Other kidney injury markers such as A1M, B2M, and cystatin C are also included in the boundary of NAP [70]. In addition, NAP is very convenient to measure in routine clinical settings because it requires only measurements of total protein, albumin, and creatinine in spot urine samples.

## 3. Clinical Significance of NAP in DKD

NAP is usually calculated as the NAP-to-creatinine ratio (NAPCR) through the following calculation:

NAPCR = urinary protein-to-creatinine ratio (UPCR) − UACR

However, there is no standard reference range or cutoff value for NAPCR. A Korean research team once set the cutoff value to 120 mg/g [14]. In some studies, NAP often refers to a condition with UPCR ≥ 150 mg/g and UACR < 30 mg/g, also called ‘isolated NAP (iNAP)’, which was generally used to emphasize the significance of NAP as a complementary DKD marker to albuminuria [71,72].

Little is known about the proportion of patients with abnormal NAP levels in DKD, especially in normoalbuminuric patients. In a single center cross-sectional study in Korea, researchers recruited 883 type 2 DM patients who had undergone both blood tests and urinary measurements simultaneously [71]. In that population, the mean eGFR was over 90 mL/min/1.73 m^2^, the prevalence of iNAP defined as UPCR ≥ 150 mg/g and the UACR < 30 mg/g, was 10.9%. In a study that analyzed 1741 subjects with stage 3 chronic kidney disease, the iNAP prevalence was 6.0% [73]. That study included all patients with or without DM and defined iNAP slightly differently as UPCR ≥ 17 mg/mmol (150.4 mg/g) and UACR < 3 mg/mmol (26.6 mg/g). In another study that included random urine samples from the United States nationwide database, the prevalence of iNAP was 10.1% [74].

As described above, NAP was composed of and correlated with various tubular markers. In addition, a study comparing electrophoretic profiles with the histological findings of renal biopsies revealed that urinary proteins other than albumin reflected tubular injury, rather than glomerular damage [75]. In line with this, a low urinary albumin to total protein ratio, which reflects the portion of NAP, was reported to be associated with tubular disease [76,77].

In 2013, a Korean research team first reported that NAP was correlated with the progression of DKD [78]. They recruited 237 type 2 DM patients with eGFR ≥ 30 mL/min/1.73 m^2^. At baseline, urinary NAPCR was positively correlated with UACR, and was significantly different even between normo-albuminuria and microalbuminuria groups. When the researchers followed those patients for a median of 29 months, NAPCR was significantly associated with an annual decline in eGFR and remained statistically associated in the multivariate regression analysis, even after adjusting for UACR, baseline eGFR, and cystatin C. In the subgroup analysis, the predictive power of NAPCR to DKD progression remained statistically significant in patients with normo-albuminuria or eGFR ≥ 60 mL/min/1.73 m^2^, suggesting that NAPCR had prognostic values for early stage DKD.

To evaluate the value of NAP as an early DKD marker, a longitudinal study which included 73 type 2 DM patients with eGFR ≥ 60 mL/min/1.73 m^2^ was conducted [79]. The mean duration of the follow-up period was 50 months, and the predictive value of NAP for DKD progression was compared with other urinary markers such as UACR, three tubular markers that included L-FABP, KIM-1, and NGAL, two proinflammatory markers that included interleukin-18 (IL-18) and YKL-40, and an intrarenal RAAS marker, angiotensin. As expected, urinary NAPCR was significantly correlated with annual eGFR decline. Surprisingly, NAPCR was the only marker that maintained its statistical association with the annual decline of eGFR in multivariate linear regression analysis which assessed all urinary markers including uACR. It was also a powerful predictor of the development of chronic kidney disease (CKD) stage 3 or greater in patients with DM.

Subsequently, based on these findings that NAP is a valuable marker for early DKD comparable to albuminuria, another study assessed the predictive value of adding NAP to albuminuria for DKD [14]. The study was a retrospective cohort study which included 325 type 2 DM patients with eGFR ≥ 30 mL/min/1.73 m^2^. Researchers defined NAP using the cutoff value of urinary NAPCR ≥ 120 mg/g and found that NAP was significantly associated with CKD progression, defined as a decline in CKD stage accompanied by a ≥ 25% decrease in eGFR from the baseline. Interestingly, in multivariate Cox regression analysis, the normoalbuminuric NAP group exhibited a high hazard ratio (HR) for CKD progression (HR = 21.82, 95% confidence interval (CI) 2.57–185.62), which was greater than the normal NAPCR albuminuric group (HR = 11.62, 95% CI 1.19–113.97) and comparable to the albuminuric NAP group (HR = 21.40, 95% CI 2.70–169.78). After assessing the concordance index (C-index) and Akaike Information Criterion (AIC) for the model fit for the Cox regression analysis, adding the categorical variable NAP to albuminuria significantly improved the model fit for CKD progression and annual eGFR decline.

Considering these results, NAP can be proposed as a useful marker for early DKD. In particular, we should pay attention to DM patients who have elevated proteinuria or NAP, even without albuminuria. In DM patients without albuminuria, NAP was not only associated with DKD, but was also associated with other comorbidities. Compared with DM patients whose UACR and UPCR were within normal limits, patients with iNAP exhibited a higher prevalence of vascular disease, such as coronary artery disease, peripheral artery obstructive disease, and ischemic stroke [71]. In that study, total proteinuria was found to have a stronger association with vascular disease than albuminuria. Furthermore, we reported that the presence of proteinuria was significantly associated with atherosclerosis, assessed by measuring carotid artery intima-media thickness, even in DM patients without albuminuria [72].

Recently, we investigated the association between urinary markers and NAFLD [80], which was also known to be a comorbidity as well as a risk factor of DM [81]. In that study, type 2 DM patients with iNAP exhibited higher scores for hepatic fibrosis. In addition, total proteinuria was significantly associated with hepatic fibrosis, whereas albuminuria tended to be correlated with hepatic steatosis, rather than fibrosis. This result is acceptable because albuminuria is known to be associated with insulin resistance [82], which is one of the fundamental pathogenic factors of NAFLD [83]. In addition, through this result, we can assume that iNAP in DM patients may reflect the progression of organ damage by other mechanisms besides insulin resistance such as hypoxia, RAAS activation, or inflammation. In the above-mentioned cross-sectional study [71], DM patients with iNAP exhibited a significantly lower secretory function of beta-cells while the index of insulin resistance was minimally altered, supporting our opinion. Previous studies of NAP in this section are summarized in Table 4.

Because NAP contains a wide range of urinary tubular injury markers, it is expected to be very sensitive in screening for early DKD when used with albuminuria, while it is estimated that the specificity might be a little lower. However, in terms of screening, it is clinically meaningful to increase the sensitivity, especially considering the convenience and low cost of the NAP measurement. In particular, NAP might be an important indicator for early DKD in DM patients without albuminuria.

In patients with increased NAP, additional studies are needed to determine whether to start the therapies for renoprotection, such as RAAS blockers, glucagon-like-1 (GLP-1) receptor agonists, and SGLT-2 inhibitors. However, those therapies were thought to be beneficial since they had favorable mechanisms for tubular injury, as well as glomerular damage. In particular, for DM patients with increased NAP, the selection of GLP-1 receptor agonists or SGLT-2 inhibitors as an antidiabetic drug would be recommended, because their direct reno-protective mechanisms, such as the suppression of inflammation and inhibition of oxidative injury [84,85,86], may also be effective for tubular injury.

## 4. Conclusions

**Figure 1:**
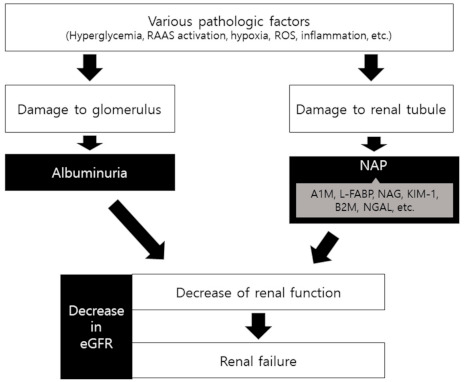
Simplified flow chart of DKD progression. Black-color boxes are the indices or markers for each step. RAAS, renin-angiotensin-aldosterone system; ROS, reactive oxygen species; NAP, non-albuminuric proteinuria; A1M, alpha-1 micro-globulin; L-FABP, liver-type fatty acid–binding protein; NAG, N-acetyl-beta-D-glucosaminidase; KIM-1, kidney injury molecule-1; B2M, beta-2 micro-globulin; NGAL, neutrophil gelatinase associated lipocalin; eGFR, estimated glomerular filtration rate.

NAP is a useful tubular marker. It contains a wide range of other tubular biomarkers and is significantly correlated with them. Previous studies have shown that NAP reflected and predicted the progression of DKD as well as the status of early stage DKD. In addition, it is convenient and inexpensive to measure. Therefore, considering the complex pathogenic mechanisms of DKD and its clinical usefulness, NAP could be suggested as a complementary marker for early stage DKD to conventional screening markers (Figure 1).

In particular, clinicians should approach DM patients without albuminuria carefully if they have elevated levels of NAPCR or UPCR. Although these patients can be categorized as a low-risk group through the conventional approach, they may actually have tubulopathy or other organ disorders such as vascular disease and liver fibrosis.

## Figures and Tables

**Table 1:** Classification of glomerulopathy of diabetic nephropathy by Renal Pathology Society.

Class	Description	Inclusion Criteria
I	Mild or nonspecific LM changes and EM-proven GBM thickening	Biopsy does not meet any of the criteria mentioned below for class II, III, or IV
		GBM > 395 nm in female and >430 nm in male individuals 9 years of age and older ^1^
IIa	Mild mesangial expansion	Biopsy does not meet criteria for class III or IV
		Mild mesangial expansion in >25% of the observed mesangium
IIb	Severe mesangial expansion	Biopsy does not meet criteria for class III or IV
		Severe mesangial expansion in >25% of the observed mesangium
III	Nodular sclerosis (Kimmelstiel–Wilson lesion)	Biopsy does not meet criteria for class IV
		At least one convincing Kimmelstiel–Wilson lesion
IV	Advanced diabetic glomerulosclerosis	Global glomerular sclerosis in >50% of glomeruli
		Lesions from classes I through III

LM, light microscopy; EM, electron microscopy; GBM, glomerular basement membrane. ^1^ On the basis of direct measurement of GBM width by EM.

**Table 2:** Classification of interstitial and vascular lesion of diabetic nephropathy by Renal Pathology Society.

Lesion	Criteria	Score
Interstitial lesions		
IFTA	No IFTA	0
	<25%	1
	25% to 50%	2
	>50%	3
Interstitial inflammation	Absent	0
	Infiltration only in relation to IFTA	1
	Infiltration in areas without IFTA	2
Vascular lesions		
Arteriolar hyalinosis	Absent	0
	At least one area of arteriolar hyalinosis	1
	More than one area of arteriolar hyalinosis	2
Presence of large vessels		Yes/No
Arteriosclerosis ^1^	No intimal thickening	0
	Intimal thickening less than thickness of media	1
	Intimal thickening greater than thickness of media	2

IFTA, interstitial fibrosis and tubular atrophy. ^1^ Score of the worst artery.

**Table 3:** Classical view of natural history of diabetic kidney disease.

Stage	Albuminuria	eGFR
I. Hyper-filtration	Possibly increased	Increased
II. Silent	Returns to normal	Returns to normal
III. Incipient	Increased	Persistent
IV. Overt	Progressed	Decreased
V. End stage renal disease	Various within morbid range	Progressed

eGFR, estimated glomerular filtration rate.

**Table 4:** Previous studies reporting clinical significance of NAP.

Index	Design	Subjects	Characteristics	Variable Correlated with the Index
NAPCR [13]	Cross-sectional	118	Type 2 DM, eGFR ≥ 60	UACR, L-FABP, KIM-1, NGAL
NAPCR [78]	Longitudinal, observational	237	Type 2 DM, eGFR ≥ 30	Baseline UACR Annual decline of eGFR during median 29 months
NAPCR [79]	Longitudinal, observational	73	Type 2 DM, eGFR ≥ 60	Annual decline of eGFR, development of CKD stage 3 or greater during median 50 months
NAPCR [14]	Longitudinal, observational	325	Type 2 DM, eGFR ≥ 30	Decline of eGFR, progression of CKD stage during median 4.3 years
Isolated NAP [71]	Cross-sectional	883	Type 2 DM, Not on RRT	(vs. normal UACR and UPCR) proportion of female, lower BMI, eGFR, glucometabolic parameters, beta cell function, vascular disease (vs. albuminuria group) higher eGFR
UPCR [72]	Cross-sectional	2047	Type 2 DM, eGFR ≥ 15,UACR<30	Carotid artery intima-media thickness
Isolated NAP [80]	Cross-sectional	1108	Type 2 DM, eGFR ≥ 15	Hepatic fibrosis

NAP, non-albumin proteinuria; NAPCR, non-albumin proteinuria-to-creatinine ratio; DM, diabetes mellitus; eGFR, estimated glomerular filtration rate; UACR, urinary albumin-to-creatinine ratio; L-FABP, liver-type fatty acid–binding protein; KIM-1, kidney injury molecule-1; NGAL, neutrophil gelatinase associated lipocalin; CKD, chronic kidney disease; RRT, renal replacement therapy; UPCR, urinary protein-to-creatinine ratio.

## Data Availability

Not applicable.
